# Cardiovascular risk factors with an emphasis on hypertension in the Mura Indians from Amazonia

**DOI:** 10.1186/s12889-018-6160-8

**Published:** 2018-11-13

**Authors:** Zilmar Augusto de Souza Filho, Alaidistânia Aparecida Ferreira, Juliano dos Santos, Karina Cardoso Meira, Angela Maria Geraldo Pierin

**Affiliations:** 1 Federal University of Amazonas, Manaus, AM Brazil; 2 University of São Paulo, School of nursing, São Paulo, SP Brazil; 3 Federal University of Rio Grande do Norte, Natal, RN Brazil

**Keywords:** Indigenous population, Cardiovascular diseases, Risk factors, Hypertension, Prevalence

## Abstract

**Background:**

The Brazilian indigenous population is currently undergoing a process of epidemiological transition regarding the occurrence of communicable diseases, malnutrition and non-communicable chronic diseases. Chronic non-infectious diseases are the most common causes of death worldwide, and hypertension is one of the main cardiovascular risk factors. Thus, the main objective of this paper was to evaluate the prevalence of cardiovascular risk factors, with an emphasis on hypertension, in the Mura Indians living in the municipality of Autazes in the northern Brazilian state of Amazonas.

**Methods:**

This cross-sectional study was conducted among 455 natives (57.8% women, 42.2 ± 16.7 years) selected by simple random sampling. Sociodemographic variables, habits and lifestyles, anthropometric data, fasting glycaemia and lipid profiles were evaluated. Blood pressure was measured with a validated automatic device. Values of *p* ≤ 0.05 were considered significant.

**Results:**

The prevalence of hypertension was 26.6%. The other cardiovascular risk factors were as follows: increased waist-hip ratio (85.1%); increased neck circumference (60.2%); increased waist circumference (48.6%); overweight (57.1%); physical inactivity (52.7%); use of alcoholic beverages (40.2%); high total cholesterol (27.5%); increased triglycerides (23.5%); smoking (20.4%); and diabetes mellitus (3.0%). In relation to non-hypertensive individuals, indigenous hypertensive individuals were (*p* ≤ 0.05) older and had a higher proportion of individuals living with partners and individuals who were retired, as well as a lower level of schooling and higher family income. The indigenous people living in urban areas had a higher prevalence of hypertension than did those living in rural areas. In relation to habits and lifestyles, hypertensive Indians had a lower prevalence of smoking, higher frequency of the use of animal fat during meal preparation, lower frequency of vegetable oil use and lower frequency of salt addition to already-prepared meals. An assessment of anthropometric variables and laboratory markers showed that the hypertensive indigenous individuals had higher values of body mass index, neck circumference, waist circumference, visceral fat, Conicity Index, and body fat than did the non-hypertensive individuals.

**Conclusion:**

The prevalence of hypertension and other important cardiovascular risk factors in the Mura Indians was high. This finding is probably due to the adoption of inappropriate habits and lifestyles.

## Background

The Brazilian indigenous population is currently undergoing a process of epidemiological transition with the occurrence of communicable diseases, malnutrition and non-communicable chronic diseases. The First National Survey of Indigenous People’s Health and Nutrition in Brazil showed that 15.8% of the indigenous population had obesity, 30.3% were overweight, and 13.2% had hypertension [1]. The changes in indigenous health are related to the processes of change in culture, habits and lifestyles and the greater access to technological resources and industrialized food products [2–5]. The origin of these changes lies in the historical process of socioeconomic and environmental changes, mainly resulting from the opening of demographic frontiers and the loss of indigenous territories [5]. As a result, indigenous peoples had more exposure to cardiovascular risk factors and cardiovascular diseases, which are currently the main cause of morbidity and mortality in the world [6].

In addition to being a public health problem, hypertension is one of the main risk factors for the development of cardiovascular diseases [7]. According to the World Health Organization’s 2014 Report, cardiovascular diseases accounted for 31% of the mortality in Brazil [8]. The prevalence of hypertension in the Brazilian indigenous population is quite varied; it is non-existent in the Yanomami Indians [9] and is prevalent in 29.5% of the indigenous reservation population of Aldeia Jaguapiru [10]. A meta-analysis review study that included 23 publications on the prevalence of hypertension among Brazilian natives reported that between 1966 and 2012, the prevalence of hypertension increased by 6.2%, with a 12% increase in the chance of an Indian presenting with hypertension in each year of the evaluation period [11]. In the indigenous populations in Brazil, other cardiovascular risk factors were also found, such as overweight [1, 12–14], increased waist circumference [15], diabetes mellitus [12, 13], hypertriglyceridemia [14, 15], increased waist-to-hip ratio [16], hypercholesterolemia [17, 18], sedentary lifestyle [19], smoking [20], and the ingestion of alcoholic beverages [21, 22].

Amazonas, one of the 27 federative units in Brazil, is the largest state, with an area of 1,559,159,148 km^2^, and its indigenous population consisted of 183,514 individuals in 2010 [23]. Three studies on cardiovascular risk factors were performed among natives in the state of Amazonas. The first study, from 1992, was with the Yanomami ethnic group from the villages of Maturacá and Maiá, in northwestern Amazonas [24]. The second study [1], conducted in 2013 and titled “The First National Survey of Indigenous People’s Health and Nutrition in Brazil”, has not yet released results stratified by ethnicity and state. The third study involved the Indians of the Upper Rio Negro and Sateré-Mawé ethnic groups who live in urban areas of Manaus, the main city of Amazonas [25].

Data from the Brazilian demographic census of 2010 [23] indicated that the indigenous population includes 896,900 individuals, representing 0.47% of the Brazilian population; of these, 36.2% lived in urban areas, and 63.8% lived in rural areas. The North Region stands out as the area with the largest indigenous population in Brazil, or 342,000 indigenous people. In this context, the Mura Indians represent the 13th largest ethnic group in Brazil, with approximately 12,479 Indians living in villages and in urban areas, due to the effect of Amazonia’s territorialisation policies in favour of extractive activities, and 7769 indigenous people live outside indigenous lands. Additionally, due to these circumstances, which differ from the Mura of the other ethnic groups studied, the indigenous people are more exposed to the substitution of Western habits and lifestyles for their culture and customs. Thus, the epidemiological transition from infectious diseases to non-communicable diseases would have greater magnitude and transcendence. Therefore, it is important to understand the health data of these populations for the creation of public policies that meet the current needs of the indigenous people. Thus, the objective of the present study was to evaluate the prevalence of cardiovascular risk factors, with an emphasis on hypertension, in Mura Indians living in the municipality of Autazes in the state of Amazonas in northern Brazil.

## Method

A cross-sectional population-based study was conducted among Mura Indians living in the city of Autazes in the state of Amazonas, located in northern Brazil. The city of Autazes is located in the central region of the state of Amazonas, far from the capital Manaus, approximately 110 km in length and 218 km inland by waterway. Autazes was chosen for its large contingent of indigenous people, composed of three ethnic groups: Apurinã, Mura and Munduruku, the largest of which is the Mura ethnic group. In the state of Amazonas, the Mura ethnic group includes 60% of the population served by the special indigenous health district Manaus. The study was carried out in two poles of the city of Autazes: Pantaleão and Murutinga.

### Participants and recruitment

The population considered for the sample calculation comprised 2676 indigenous people, distributed between urban areas (*n* = 1059) and rural areas (*n* = 1617) of the city of Autazes. The sample size was calculated based on the prevalence of hypertension of 30% [10] and the loss rate, represented as 3.2% (*n* = 15) of the sample initially estimated, resulting in 455 indigenous adults (234 residents in rural areas and 221 in urban areas). The absence of the Indians at the health unit (Polo Base) for data collection indicated the need for the researchers to return to individuals’ homes at later times, and after the third unsuccessful attempt to come to their homes, the participants’ absence was recorded as sample loss.

The Indians were selected by simple random sampling, based on data from the indigenous registration system of the city of Autazes. The inclusion criteria for the indigenous participants were as follows: living in the villages of Pantaleão and Murutinga, being 18 years of age or older, having the *Registro Administrativo de Nascimento Indígena* (RANI - Indian Birth Registry) issued by Fundação Nacional do Índio (FUNAI - National Indian Foundation) and speaking Portuguese. Pregnant women were not included. All participants signed the informed consent forms.

### Procedures and setting

Data collection was performed by trained nurses at the indigenous health units (Pantaleão and Murutinga) and included casual blood pressure measurement, fasting capillary glycaemia, lipid profile, physical examination and interviews.

During the interviews with the indigenous people, the following data were collected. First, sociodemographic evaluations were conducted and included the collection of data on sex, age, marital status, schooling, family income, retirement and socioeconomic classification. Socioeconomic classifications were determined according to the “Brazilian Economic Classification Criteria [26], which considers participants” household conditions (number of bathrooms, water source, type of paving of the street where the domicile is located, and the number of the domestic maids), quantity of durable consumer goods, household appliances and electronics, and levels of education. From these data, the person is classified into one of the following categories: A, B1, B2, C1, C2, and D-E. The monthly household income was calculated based on the minimum wage, which, at the time of data collection, was 880 reals, which corresponded to USD $237.80. Second, personal history was assessed using self-reported data on hypertension, heart problems, stroke, diabetes and dyslipidaemia. Third, with regard to habits and lifestyles, smoking was evaluated by self-report. Participants were asked whether they were smokers (yes/no/ex-smokers). Smokers and ex-smokers were asked, respectively, “How long have you smoked?” and “How long has it been since you have stopped smoking?” Smokers were also questioned about the number of packs per day they smoked (less than one/one/two/more than two/do not know how to answer). In addition, alcohol consumption was assessed using the Alcohol Use Disorders Identification (AUDIT) instrument, validated for the Portuguese language [27], which is aimed at the early detection and identification of risk groups and the screening of problematic alcohol use and its consequences in the previous year to develop interventions, both in clinical samples and in the general population. The AUDIT consists of 10 questions that explore the characteristics, dependence and problems related to alcohol use. The score ranges from 0 to 40, but a score of eight or more indicates the need for a more specific diagnostic investigation. We classified participants according to AUDIT if they were at risk (yes) or at not at risk (no) for alcohol abuse; thus, we used this variable as the outcome. The first AUDIT question is about the frequency of consumption and is answered on a scale ranging from 0 (never) to 4 (four or more times per week). The answer “never” classifies the participant as not consuming alcohol.

The level of physical activity was assessed by the International Physical Activity Questionnaire (IPAQ, short version) [28]. Dietary habits were evaluated using an instrument adapted from the *Inquérito Nacional de Saúde e Nutrição dos Povos indígenas* (National Survey of Health and Nutrition of Indigenous Peoples) [29], and it considered the origin of the food, the use of fat in food preparation, the type of fat used, salt consumption and the use of sugar in food preparation, along with the weekly consumption of other food items in indigenous daily life. The Indians’ food consumption frequency scores were calculated by assigning the following values to each response: 1 point (almost never/never); 2 points (1 to 2 days); 3 points (3 to 4 days); 4 points (5 to 6 days); and 5 points (every day). The consumption values assigned to each food category ranged from 1 to 5 points, and the higher the score, the greater the frequency of food consumption (almost never/never = 1 to 2 days = 2 points; 3 to 4 days = 3 points; 5 to 6 days = 4 points; every day = 5 points). Based on these values, a weekly average consumption score was calculated for the consumption of fresh foods (fruits and vegetables), processed products (canned feijoada, canned sardines, salchicha, ham and bacon) and high-calorie industrialized foods (cakes, sweets, processed juices, soft drinks and chocolates).

Fourth, an anthropometric evaluation was conducted. Body weight was measured with a digital bioimpedance scale with a maximum capacity of 150 kg, and height was measured using a stadiometer with a scale of 20 to 200 cm and a precision of 0.1 cm, fixed on a rigid wall. Neck and waist circumferences were measured with an inelastic tape measure. The circumference of the neck was considered altered in men for values ≥37 cm and in women for values ≥34 cm [30]. Waist circumference was considered to be altered for values ≥102 cm in men and for values ≥88 cm in women [31]. The percentage of body fat and the percentage of visceral fat were evaluated by electrical bioimpedance. The assessment of body fat varied according to age and sex [32]. The levels of visceral fat were categorized as normal (≤ 9%), high (10–14%) and very high (≥15%) [33]. Fifth, blood pressure measurement was performed with a validated automatic device [34], with a cuff sized appropriately to the circumference of the arm. The Indian participants were seated comfortably, their feet flat on the ground and their left arm resting on their heart, and their measurements were taken after ten minutes of rest. Before their blood pressure was measured, the participants were instructed to empty the bladder and to confirm that they had not drunk alcohol or coffee and had not smoked up to 30 min before. Three blood pressure measurements were performed, and the mean of the last two measurements was used in the data analyses. Hypertension was defined as systolic blood pressure ≥ 140 and/or diastolic blood pressure ≥ 90 mmHg; alternatively, hypertension could be self-reported (referred) when indigenous participants reported having been diagnosed with hypertension by a physician or a nurse or when they were taking anti-hypertensive medicine, independently of the blood pressure values measured in the interview. Hypertension was classified in stages following the recommendations of the VII Brazilian Guidelines on Hypertension [7]. Sixth, the evaluations of capillary glycaemia and lipid profiles were performed after an eight-hour fast with a portable digital device. Diabetes mellitus was considered for glycaemia values ≥126 mg/dL, high cholesterol ≥240 mg/dL and triglycerides ≥200 mg/dL [35, 36].

### Statistical analyses

In the statistical analysis, the qualitative variables were presented in absolute (n) and relative (%) frequencies, and the quantitative variables were presented using the means and standard deviations (SD). The existence of an association between each independent variable and hypertension (yes/no) was assessed using Pearson’s chi-square test or Fisher’s exact test. The differences between the averages of the quantitative variables were evaluated by Student’s t-test or the Mann-Whitney U test, according to the normality of the distribution of the studied variable. In the present study, we sought to evaluate the prevalence of factors associated with cardiovascular risk in the Mura ethnic group and their relation to hypertension. Because the design of the study in question is cross-sectional, in which exposure data and independent variables are collected at the same time, it was not possible to determine the temporality between these variables, that is, cause-and-effect relationships. Thus, we decided not to perform the multiple analysis, since the associations found, especially between habits and lifestyles and hypertension, could be related to reverse causality and survival bias.

### Ethics

Data were collected from January to March 2016, after approval by the Ethics Committee of the School of Nursing, University of São Paulo (no. 801.832/2014), of the National Commission for Research Ethics (CONEP no. 1,199,398/2015) and the National Indian Foundation (127/AAEP/PRES/2015). None of the authors have any competing interests. After all the explanations about the procedures of the study were provided to participants, all indigenous individuals who accepted to participate signed the free and informed consent form.

## Results

The prevalence of hypertension in the studied population of Indians was 26.6% (95% CI 22.5–30.7%). The data in Table 1 show that the majority of the sample was female; lived without a partner; had a monthly family income lower than minimum wage, corresponding to USD $237.80; had some type of social benefit; and belonged to economic class D or E (86.4%). The mean age of the studied population was 42.1 years, and the participants’ educational level was low, as only 27.3% had completed high school. For place of residence, 51.4% were from rural areas, and the rest were from urban areas.

**Table 1:** Sociodemographic variables and hypertension in the Mura indigenous individuals, Autazes - Amazonas, Brazil

Variables	Hypertension			*p*-value
	No (*n* = 334)	Yes (*n* = 121)	Total Sample (*N* = 445)	
	N (%)	N (%)	N (%)	
Sex				0.521^a^
Female	190 (56.9)	73 (60.3)	263 (57.8)	
Male	144 (43.1)	48 (39.7)	192 (42.2)	
Age (years), mean (SD)	37.91 (14.7)	53.66 (16.63)	42.1 (16.74)	< 0.001^b^
Marital status				0.001^c^
Without partner	258 (77.2)	73 (60.3)	331 (72.7)	
With partner	76 (22.8)	48 (39.7)	124 (27.3)	
Education				< 0.001^c^
Illiterate/elementary I (1st–5th ^y^ear)	116 (34.7)	71 (58.6)	187 (41.1)	
Incomplete elementary II (6th–9th year)	62 (18.6)	15 (12.4)	77 (16.9)	
Incomplete high school	43 (12.9)	6 (5.0)	49 (10.8)	
Complete high school	100 (29.9)	24 (19.8)	124 (27.3)	
Complete higher education	13 (3.9)	3 (2.5)	16 (3.5)	
Post-graduate studies	–	2 (1.7)	2 (0.4)	
Monthly family income (minimum salary)^*^				0.036^c^
< 1	133 (39.8)	38 (31.4)	171 (59.4)	
1–2	147 (44.0)	69 (57.1)	216 (34.2)	
≥ 3	54 (16.2)	13 (11.5)	68 (6.4)	
Receives retirement salary				< 0.001^c^
Yes	68 (20.4)	52 (43.0)	120 (26.4)	
No	266 (79.6)	69 (57.0)	335 (73.6)	
Socioeconomic stratum				0.078^a^
B2	3 (0.9)	3 (2.5)	6 (1.3)	
C1-C2	35 (10.5)	21 (17.4)	56 (12.3)	
D-E	296 (88.6)	97 (80.1)	393 (86.4)	
Place of residence				
Rural area	183(78.2)	51(21.8)	234(51.4)	0.020^c^
Urban area	151(68.3)	70(31.7)	221(48.6)	
Social programme benefit				< 0.001^c^
Yes	221(66.2)	53(43.8)	274(60.2)	
No	113(33.8)	68(56.2)	181(39.8)	

* Minimum salary 880 reais = 237.8 dollars

^a^Fisher’s exact test

^b^Student’s t-test

^c^Pearson’s chi-square test

Regarding habits and lifestyles, slightly more than half of the sample [52.7% (95% CI: 48.2–57.3%)] was sedentary; 40.2% (95% CI: 35.7–44.7%) reported consuming alcoholic beverages; and 20.4% (95% CI: 16.7–24.1%) were smokers. With regard to food preparation, most of the participants used vegetable oil [98.9% (95% CI: 97.6–99.6%)] and salt [99.6% (95% CI: 98.4–99.9%)], in addition to adding salt to already-prepared meals [51.2% (95% CI: 46.1–55.9%)] and consuming sugar [98.9% (95% CI: 97.6–99.6%)] (Table 2).

**Table 2:** Habits, lifestyles and personal background in the Mura indigenous individuals. Autazes - Amazonas, Brazil

Variables	Hypertension			*p*-values
	No (n = 334)	Yes (n = 121)	Total Sample (N = 445)	
	N (%)	N (%)	N (%)	
Smoking				
Yes	78 (23.4)	15 (12.4)	93 (20.4)	< 0.001^a^
No	256 (76.6)	106 (87.6)	362 (79.6)	
Ex-Smoker	0 (0.0)	0 (0.0)	0 (0.0)	
Alcohol intake				
Yes	141 (42.2)	42 (34.7)	183 (40.2)	0.161^a^
No	193 (57.8)	79 (77.0)	272 (59.8)	
Physical activity				0.457^a^
Sedentary/irregularly active	180 (53.9)	60 (49.6)	240 (52.7)	
Active	154 (46.1)	61 (50.4)	215 (47.3)	
Fat in food preparation				
Olive oil	10 (3.0)	4 (3.3)	14 (3.1)	1.000^a^
Animal fat or lard	17 (5.1)	15 (12.4)	32 (7.0)	0.012^a^
Vegetable oil	333 (99.7)	117 (96.7)	450 (98.9)	0.019^b^
Add salt to meals				
Yes	181 (54.2)	52 (43.0)	233 (51.2)	0.043^a^
No	153 (45.8)	69 (57.0)	222 (48.8)	
Personal background				
Heart problems	12 (3.6)	18 (14.9)	30 (6.6)	< 0.001^a^
Stroke	4 (1.2)	11 (9.1)	15 (3.3)	< 0.001^b^
Diabetes mellitus	8 (2.4)	15 (12.4)	23 (5.1)	< 0.001^a^
Dyslipidaemias	32 (9.6)	35 (29.2)	67 (14.7)	< 0.001^a^

^a^Fisher’s exact test

^b^Pearson’s chi-square test

Anthropometric and laboratory data (Table 4) showed that the majority of the studied Indians had increased neck circumference [60.2% (95% CI: 55.7–64.7%)], were overweight [57.1% (95% CI: 46.7–67.6%)] and had high body fat [69.3% (95% CI: 65.0–73.5%)]. Nearly half had increased waist circumference [48.6% (95% CI: 44.0–53.2%)]. Cholesterol levels were elevated by 27.5% (95% CI: 21.3–33.7%) and triglycerides by 23.5% (95% CI: 19.6–27.4%), and 3.0% of the sample presented diabetes mellitus (95% CI: 1.8–5.1%).

In relation to non-hypertensive individuals, the indigenous hypertensive individuals were (*p* ≤ 0.05) older, and a higher proportion of individuals lived with their partners, were retired, and had lower levels of schooling and higher family income. The indigenous people living in urban areas had a higher prevalence of hypertension than did those living in rural areas (Table 1). With regard to personal history, hypertensive patients reported (*p* ≤ 0.05) a greater frequency of heart problems, stroke, diabetes mellitus and dyslipidaemia. With regard to habits and lifestyles, the hypertensive patients had a lower prevalence of smoking, a higher frequency of the use of animal fat in the preparation of meals, a lower frequency of vegetable oil use, and a lower frequency of the addition of salt to already-prepared meals (Table 2).

Regarding the score for average food consumption during the week, there was a low frequency of the consumption of natural foods, both in hypertensive and non-hypertensive Indians [1.74 (SD = 1.19) vs 1.77 (SD = 1.15)]. Although the average consumption of hypertensive Indians was higher than that of non-hypertensive Indians, this difference was not statistically significant (*p* = 0.08). The mean score for the consumption of processed foods (canned feijoada, canned sardines, salchicha, ham and bacon) was the same among hypertensive and non-hypertensive Indians (1.30 (SD = 0.66) vs 1.30 (SD = 0.68), *p* = 0.08). Notably, the highest mean score was for hypercaloric processed foods (cakes, sweets, soft drinks and chocolates), both in hypertensive and non-hypertensive subjects, being higher in hypertensive Indians than in non-hypertensive Indians, and this difference was not statistically significant [1.96 (SD = 1.23) vs 1.85 (SD = 1.23), *p* = 0.20)]. The origin of food draws the attention of the greater proportion of indigenous people who buy their food independent of their hypertension status and who, for the most part, do not grow food at home. A higher prevalence of food originating from the fishery and from collection was found among non-hypertensive natives than among hypertensive natives, and this difference was statistically significant (Table 3).

**Table 3:** Origin of foods and hypertension in the Mura indigenous individuals. Autazes - Amazonas, Brazil

Variables	Hypertension					
	No (n = 334)		Yes (n = 121)		Total sample (*N* = 455)	*p*-values
	N	%	N	%	N (%)	
Food cultivation						
Yes	166	49.7	57	47.1	223 (49.0)	0.672^a^
No	168	50.3	64	52.9	232 (51.0)	
Hunting/fishing						
Yes	228	68.3	59	48.8	287 (63.1)	< 0.001^a^
No	106	31.7	62	51.2	168 (36.9)	
Food collection/home-breeding						
Yes	70	21.0	26	21.5	96 (21.1)	0.897^a^
No	264	79.0	95	78.5	359 (78.9)	
Food purchase						
Yes	331	99.1	121	100.0	452 (99.3)	0.569^a^
No	3	0.9	0	0	3 (0.7)	
Receive food donation						
Yes	20	6.0	5	4.1	25 (5.5)	0.641^a^
No	314	94.0	116	95.9	430 (94.5)	

^a^Pearson’s chi-square test

The assessment of the anthropometric variables and laboratory markers showed that hypertensive indigenous individuals (*p* ≤ 0.05) had higher values for body mass index, neck circumference, waist circumference, visceral fat, Conicity Index, and body fat than did non-hypertensive individuals. Triglyceride levels and total cholesterol were in the high range, and there was a higher prevalence of diabetes mellitus (Table 4).

**Table 4:** Anthropometric variables, laboratory markers and hypertension in Mura indigenous individuals. Autazes - Amazonas, Brazil

Variables	Hypertension			*p*-values
	No (n = 334)	Yes (n = 121)	Total sample (N = 455)	
	N (%)	N (%)	N (%)	
BMI (kg/m^2^), mean (SD)	25.82 (4.38)	28.94 (5.07)	26.65 (4.77)	< 0.001^a^
Neck circumference				< 0.001^b^
Normal	151 (45.2)	30 (24.8)	181 (39.8)	
Increased	183 (54.8)	91 (75.2)	274 (60.2)	
Waist circumference				< 0.001^b^
Normal	203 (60.8)	31 (25.6)	234 (51.4)	
Increased	131 (39.2)	90 (74.4)	221 (48.6)	
Body fat percentage				< 0.001^b^
Low	8 (2.4)	4 (3.3)	12 (2.6)	
Normal	111 (33.2)	17 (14.0)	128 (28.1)	
High	215 (64.4)	100 (82.6)	315 (69.3)	
Visceral fat				< 0.001^b^
Normal	255 (76.3)	58 (47.9)	313 (68.8)	
High	79 (23.7)	63 (52.1)	142 (31.2)	
Conicity index	1.25 (0.07)	1.32 (0.05)	1.27 (0.08)	< 0.001^a^
Triglycerides (mg/dL)	151.5 (99.8)	196.7 (111.3)	163.5 (104.7)	< 0.001^b^
Desirable	219 (65.6)	51 (42.1)	270 (59.3)	< 0.001^b^
Borderline	49 (14.7)	29 (24.0)	78 (17.1)	
High	56 (16.8)	37 (30.6)	93 (20.5)	
Very high	10 (3.0)	4 (3.3)	14 (3.1)	
Total cholesterol (mg/dL)	183.7 (28.6)	197.5 (39.0)	187.4 (32.2)	< 0.001^a^
Desirable	256 (76.6)	74 (61.2)	330 (72.5)	0.001^a^
Borderline	58 (17.4)	27 (22.3)	85 (18.7)	
High	20 (6.0)	20 (16.5)	40 (8.8)	
Glycaemia (mg/dL)	68.9 (20.0)	78.5 (30.2)	71.4 (23.5)	< 0.001^a^
Normal	327 (97.9)	111 (91.7)	438 (96.3)	0.008^a^
Decreased glucose tolerance	1 (0.3)	2 (1.7)	3 (0.7)	
Diabetes mellitus	6 (1.8)	8 (6.6)	14 (3.0)	

^a^Student’s t-test

^b^Pearson’s chi-square test

BMI Body mass index

## Discussion

The findings from the present study showed that Indian participants with hypertension had a high prevalence of hypertension, increased neck circumference, elevated quantities of body fat, sedentary lifestyles, increased waist circumference, and elevated cholesterol and triglycerides. These findings may be associated with the adoption of Western habits and lifestyles by the Mura Indians, due to the loss of their territories from the policies of expansion of agricultural frontiers and extractivism in the northern region of Brazil [1–5].

Notably, a sedentary lifestyle, overweight and obesity, dyslipidaemia, and hypertension [1, 4, 6] were the most prevalent cardiovascular risk factors among the Mura Indians (52.8%); this finding was higher than that observed between the Kaingang and Guaraní natives (45.3%) [17] and in a data survey from the general population of all Brazilian capitals (46.0%) [37]. The Mura Indians have adopted a sedentary lifestyle, mainly due to the ease of access to technology. Previously, they paddled through rivers in canoes, and they are now travelling in motor boats. Another alarming finding was the high intake of alcoholic beverages (40.2%), which was a higher frequency than that observed among indigenous people in the Jaguapiru village (31.1%) [10], in the Kaingang Indians (29.9%) [22] and among Guaraní, Kaiowá and Terena natives [14].

The present study showed that 26.6% of the Indians studied from the Mura ethnic group of the city of Autazes, Amazonas, Brazil, had hypertension. The prevalence among the Mura Indians was higher than that of other studies with indigenous populations, including the Xavante (17.2–17.5%) [16] and Tupinikim (20.8%) Indians [20], and close to that observed among the Kaingang Indians (26.8%) [18] and the indigenous people of the Jaguapiru village (29.5–29.7%) [10, 12]. The variations observed in the prevalence of hypertension may be related to different habits and lifestyles [15], disparities in the criteria for the definition of hypertension [16] and proximity to urban areas [7, 16, 22]. The proximity to urban areas seems to be the most plausible hypothesis. The findings of the present study showed higher prevalence among indigenous people in urban areas than among those in rural areas. The proximity and the contact with urban areas expose the natives to the adoption of unhealthy habits and lifestyles. Thus, the lowest prevalence was observed among the Xavante and Tupinikim Indians, which is probably attributed to their residing in indigenous reserves, while the highest prevalence was observed in ethnicities close to urban settlements [16, 19]. A systematic review with a meta-analysis of publications from 1980 to 2010 examining the prevalence of hypertension in the general population of Brazil showed a decrease in the prevalence of hypertension, from 36.1 to 28.1% [38]. Thus, the 26.6% finding of the Mura Indians is close to the findings of data from population-based studies in Brazil. This prevalence of hypertension in the Mura Indians reinforces, once again, the relationships among hypertension; the process of accelerated urbanization; the progressive increase in life expectancy; the changes in dietary patterns; the excessive consumption of foods rich in saturated fat; and the risk factors for hypertension, diabetes and obesity.

We also highlight the probable influence of the socioeconomic context, with a predominance of low-income individuals, and the fact that the majority of the Indians belong to the less-favoured classes (D-E). In the sample studied, the majority of Mura Indians (60.2%) reported participating in some direct income distribution programme by the federal government, especially the Bolsa Família Program (97.8%). Researchers investigating other ethnicities also observed this phenomenon, as among the Terena indigenous people, with 71.4% of the families included in a government social programme [39]; among the Guaraní ethnic group from Dourados, Mato Grosso do Sul, 84.2% of the indigenous families benefited from the *Bolsa Família* (Family Allowance) programme [40]. Often, social benefits are the main and only source of income for indigenous families. Indigenous people are considered to have greater social vulnerability due to the high percentage of families living in poverty and extreme poverty, which may contribute to worsening health conditions.

The results showed that the hypertensive Indians had a higher mean age than did the non-hypertensive subjects. This finding was expected because there is a direct and linear association between ageing and the prevalence of hypertension. The prevalence of hypertension was 68% in a meta-analysis review study that included 13,978 elderly people in Brazil [41]. Increasing age was also associated with hypertension in a study with indigenous people of the Guaraní, Kaiowá and Terena ethnic groups of the Jaguapiru village [10]. The results were expected to show that hypertension, a non-transmissible chronic disease, was associated with lifetime exposure to modifiable and intermediate risk factors for cardiovascular diseases.

The mean body mass index in the Mura Indians was high (26.65), and there was a statistically significant difference between the hypertensive and non-hypertensive Indians. The positive association between hypertension and body weight gain and obesity is a reality in non-indigenous [42–44] and indigenous [12, 18, 20] populations, because these modifiable risk factors act by increasing blood volume as well as decreasing peripheral vascular resistance, favouring an increase in systemic blood pressure. In addition, they may also cause increased ventricular filling pressure, which may result in increased vessel wall stress, diastolic dysfunction, and left ventricular hypertrophy, resulting in compromised cardiac work and the promotion of early signalling mechanisms for changes cellular and atheroma formation, thus bypassing this hypothesis. Among these variables, nutritional assessment, hypertensive Indians showed higher values for neck circumference; waist-hip ratio; conicity index; body age in relation to the actual age; and the percentage of body fat. The evaluation of all these anthropometric parameters in Brazilian indigenous peoples is an unprecedented fact.

Regarding food consumption, no differences were observed in the average food frequency score among hypertensive and non-hypertensive natives. However, a low frequency of in natura consumption was observed in both groups, as well as an intermediate score in the consumption of industrialized foods in hypertensive and non-hypertensive Indians. However, in relation to the origin of food, the Indians predominantly bought their food and reported a lower proportion of food that was collected, home-grown or from home-raised animals. These findings signal a transition in the food patterns of the natives in Brazil, constituting a critical scenario in the emergence of cardiovascular risk factors through the presence of excess weight, sedentary lifestyle, hypertension, diabetes, dyslipidaemias, factors directly related to these characteristics, and changes in the food patterns of these Indians, which was confirmed in the present study. Additionally, the scarcity of published studies on food consumption among indigenous populations of Brazil or specific ethnic groups.

The transition of food patterns can be related to changes in choice and the ability to obtain food that occurred in this ethnic group, due to the process of expansion of agricultural frontiers, causing individuals within this group to lose their territories and migrate to urban areas without the possibility of growing food or raising animals. In support of this hypothesis, we found a higher prevalence of hypertension among indigenous people living in urban areas, who consume less food through collecting and fishing than do non-hypertensive individuals. This finding is corroborated when the origin of the food is evaluated by urban and rural areas, since there was a significant difference (*p* ≤ 0.05) between the groups of indigenous people in rural areas compared to those in urban areas regarding food and/or animal husbandry at home, the collection of farmed and/or home-grown animals, food purchased by hunting and/or fishing, and the receipt of basic food baskets. Therefore, the results indicate that the indigenous people of rural areas had a higher availability of natural resources for hunting and fishing activities with the possibility of acquiring food than in the urban areas where the Mura Indians live; these areas present more difficulties in accessing food and a lower availability of natural resources required to purchase food, such as areas available for planting; a direct source of in natura foods (vegetables, fruits and carbohydrates) from the field; forest areas for the practice of hunting; and material resources available for fishing. Such as canoes, oars and outboard motors. Additionally, the urbanization of the city of Autazes may have a direct influence on the availability and abundance of game animals, such as tapirs, pacas, armadillos, and catitus, with effects on the availability of fish in the rivers of the region.

In this sense, an ethnographic study conducted from 2013 to 2014 on the changes in the eating habits of the Akwen Xerente Indians of the villages Porteira and Funil in the state of Tocantins, indicated that cultural changes, changes in livelihoods with the decrease in the insertion in the labour market, the provision of social benefits and the addition of technological resources through the distribution of the electric network in the villages made possible direct changes in the alimentary habits of these Indians. Evidence of these changes in dietary habits was identified by the presence of various types of industrialized food available for commercialization in Akwen Xerente indigenous villages, such as bottled and artificial juices, instant noodles, soybean oil, margarine, packaged biscuits, and powdered chocolate [45].

There were no differences in the consumption of alcohol between hypertensive and non-hypertensive Indians. However, there was a high consumption prevalence (40.2%), which was more than double that in the Brazilian population in 2013 (16.4%) [37]. In Brazil, the marketing of alcoholic beverages on indigenous lands is prohibited. However, fermented beverages made from tubers, which are typical of indigenous cultures such as the Caxiri, Aluah and Tarubá, have been replaced by distilled beverages as a consequence of the expropriation of lands, the reduction and exploitation of indigenous territories, self-sustainability and migration to urbanized areas, where the access to and marketing of alcoholic beverages is poorly controlled. In this context, alcoholism has been linked to cases of homicide, suicide, violence among groups, incest, sexual abuse and rape, and an increase in the mortality rate within indigenous areas in different Brazilian states [22].

Other important cardiovascular risk factors observed in this population were diabetes mellitus and glucose intolerance. Decreased glucose tolerance or pre-diabetic state refers to subjects who present glycemia from 140 to 199 mg / dL after 2 h in the oral glucose tolerance test (OGTT) [12].

The occurrence of diabetes mellitus and glucose intolerance contributes to vascular injury and affects renal function, favouring blood pressure elevation. In addition, the personal history of diabetes mellitus, independent of other variables, classifies the individual as having a high cardiovascular risk. Among the Mura Indians studied, 5.1% reported a history of diabetes mellitus, and 3.0% presented plasma glucose levels compatible with diabetes. These findings were higher than the prevalence observed among indigenous Guaraní (1.5%), Tupinkim (4.2%) [20] and Khisêdjê (3.8%) Indians [19] and were slightly below that observed among indigenous people in the village of Jaguapiru (5.8%) [10]. Although the prevalence of diabetes among the Mura Indians was lower than that observed in the non-indigenous population, this rate was higher than those pertaining to other ethnicities. Thus, this finding reflects the incorporation of new habits and food products, such as soft drinks, sweet drinks, ice cream, and sweets, and the high frequency of sugar utilization in food preparation.

In indigenous daily life, smoking may be related to beliefs or rituals. The prevalence of smoking among the Mura Indians was 20.4%, with a lower rate of smoking among hypertensive Indians. Among the Kaiowá, Guaraní and Terena Indians, the prevalence of smoking was 19.0% [14], while that among the Tupinikim Indians (60.4%) [20] and Kaingang and Guaraní Indians (44.0%) was much higher [17]. Although cigarette smoking among indigenous people is strongly influenced by historical and cultural manifestations, the high prevalence observed is of concern, since it is related to cardiovascular events and lesions of target organs, in addition to other non-transmissible chronic diseases. The lower prevalence of smoking observed in hypertensive Indians could be related to changes in habits and lifestyle after the diagnosis of the disease. However, in this population, the absence of ex-smokers was observed, which is not consistent with this hypothesis. Hypertension is a chronic multifactor non-communicable disease, and in the study population, other risk factors, such as measures associated with nutritional assessment and elevated triglyceride and cholesterol levels, were more prevalent in hypertensive patients

### Limitations of the research

The limitations of the present study are related to the transversal design, which does not allow for the establishment of cause-and-effect relationships. The cut-off points of the adopted anthropometric components were the same as those used for non-indigenous populations. Depending on the race and/or ethnic identification, it is not always advisable to adopt diagnostic criteria for the non-indigenous population; however, considering the absence of specific cut-off points for indigenous populations, the accepted criteria for the general population were considered. Another limitation may be the use of reagent strips to assess blood glucose, cholesterol, and triglycerides, the results of which may be less accurate than those of measurements using blood samples. However, notably, the blood pressure measurement was performed with a validated automatic device, thus reducing errors caused by the observer using the auscultatory technique. Three measures of blood pressure, and the fact that they had been performed by nurses, can soften the phenomenon of the white-coat hypertension. Additionally, this is the first study to evaluate hypertension and other cardiovascular risk factors in Mura Indians in Brazil.

## Conclusions

In conclusion, the Mura Indians presented a high prevalence of cardiovascular risk factors, especially hypertension. The findings show an epidemiological and nutritional transition, which may allow for the increase in risk factors for chronic non-communicable diseases. In light of the findings, it is important to establish health policies with a focus on the prevention of cardiovascular risk factors and health education strategies for adopting healthier habits and lifestyles. Thus, the multidisciplinary work of the health team regarding attention to the natives is necessary to meet the actual needs of these populations and to modify the profile of morbimortality resulting from the epidemiological transition that they experience.

## Abbreviations

BMI: Body mass index
CI: Confidence intervals
CONEP: Comissão Nacional de Ética em Pesquisa, National Commission for Research Ethics
FUNAI: Fundação Nacional do Índio; National Indian Foundation
PR: Prevalence ratio
RANI: Indian Birth Registry
SD: Standard deviation

## References

[1] Coimbra CE, Santos RV, Welch JR, Cardoso AM, Souza MC, Garnelo L (2013). The first National Survey of indigenous People’s health and nutrition in Brazil: rationale, methodology, and overview of results. BMC Public Health.

[2] Cardoso AM, Mattos IE, Koifman RJ (2001). Prevalence of risk factors for cardiovascular disease in the Guaraní-Mbyá population of the state of Rio de Janeiro. Cad Saude Publica..

[3] Gimeno SGA, Rodrigues D, Canón EN, Lima EES, Schaper M, Pagliaro H (2009). Cardiovascular risk factors among Brazilian Karib indigenous peoples: upper Xingu, Central Brazil, 2000–3. J Epidemiol Community Health.

[4] Rocha TES, Silva RP, Nascimento MM (2016). Changing dietary habits among Akwen Xerente. Rev Esc Enferm USP..

[5] Basta PC, Orellana JDY, Arantes R, Garnelo L, Pontes AL (2012). Perfil epidemiológico dos povos indígenas no Brasil: notas sobre agravos selecionados. organizadoras. Saúde indígena: uma introdução ao tema.

[6] Go AS, Mozaffarian D, Roger VL, Benjamin EJ, Berry JD, Blaha MJ, Dai S (2014). Executive summary: heart disease and strokestatistics - 2014update: areportfrom theAmericanHeartAssociation. Circulation.

[7] VII Brazilian Guidelines on Hypertension. Arq Bras Cardiol 2016; 107(supl.3):1–83. 10.5935/abc.20160151.

[8] Riley L, Cowan M (2014). World Health Organization noncommunicable diseases country profiles.

[9] Mancilha-Carvalho J, De J, NAS S (2003). The Yanomami Indians in the INTERSALT study. ArqBrasCardiol.

[10] Oliveira GF, Oliveira TRR, Ikejiri AT, Andraus MP, Galvao TF, Silva MT (2014). Prevalence of hypertension and associated factors in an indigenous Community of Central Brazil: a population-based study. PLoSOne.

[11] Souza Filho ZA, Ferreira AA, Santos B, Pierin AMG (2015). Hypertension prevalence among indigenous populations in Brazil: a systematic review with meta-analysis. Rev Esc Enferm USP..

[12] Oliveira GF, Oliveira TRR, Rodrigues FF, Corrêa LF, Ikejiri AT, Casulari LA (2011). Prevalence of diabetes mellitus and impaired glucose tolerance in indigenous people from Aldeia Jaguapiru. Brazil. Rev Panam Salud Publica..

[13] Port Lourenço Ana Eliza, Ventura Santos Ricardo, Orellana Jesem D. Y., Coimbra Carlos E. A. (2008). Nutrition transition in Amazonia: Obesity and socioeconomic change in the Suruí Indians from Brazil. American Journal of Human Biology.

[14] Oliveira GF, Oliveira TR, Ikejiri AT, Galvao TF, Silva MT, Pereira MG (2015). Prevalence of obesity and overweight in an indigenous population in Central Brazil: a population-based cross-sectional study. Obes Facts.

[15] Tavares FG, Coimbra CEA, Cardoso AM (2013). Blood pressure levels of Suruí indigenous adults in Rondônia. Brazil Cien Saude Colet.

[16] FabbroAL D, Franco LJ, Silva AS, Sartorelli DS, Soares LP, Franco LF (2014). High prevalence of type 2 diabetes mellitus in Xavante Indians from Mato Grosso, Brazil. Ethn Dis.

[17] Rocha AK, Bós AJ, Huttner E, Machado DC (2011). Prevalence of metabolic syndrome in indigenous people over 40 years of age in Rio Grande do Sul. Brazil Rev Panam Salud Publica.

[18] Anjos HNK, Toledo MJO, Mota LT, Previdelli ITS, Anjos AF, Saruhashi TR (2011). Prevalence of metabolic syndrome among Kaingang native Americans in southern Brazil. Braz Arch Biol Technol.

[19] Santos KM, Tsutsui MLS, Galvão PPO, Mazzucchetti L, Rodrigues D, Gimeno SGA (2012). Degree of physical activity and metabolic syndrome: a cross-sectional study among the Khisêdjê group in the Xingu Indigenous Park. Brazil Cad Saude Publica.

[20] Meyerfreund D, Gonçalves CP, Cunha RS, Pereira AC, Krieger JE, Mill JG (2009). Age-dependent increase in blood pressure in two different native American communities in Brazil. J Hypertens.

[21] MLP S, Deslandes SF, Garnelo L (2010). Ways of life and ways to drink of young indigenous in a transformation context. Cien Saude Colet.

[22] Ferreira AA, Souza-Filho ZA, Gonçalves MJF, Santos J, Pierin AMG. Relationship between alcohol drinking and arterial hypertension in indigenous people of the Mura ethnics, Brazil. PLoSOne. 2017;12(8):e0182352. doi: 10.1371. 10.1371/journal.pone.0182352 PMC5544198 28777805

[23] Brasil. Instituto Brasileiro de Geografia e Estatística (IBGE). Censo Demográfico 2010: características gerais dos indígenas. Resultados do universo. Rio de Janeiro, RJ: IBGE, 2010.

[24] Carvalho JJM, Carvalho JV, Lima JAC, Silva NAS (1992). Ausência de Fatores de Risco de Doença Coronária em Índios Yanomami e Influência da Aculturação na Pressão Arterial. Arq Bras Cardiol.

[25] Toledo NN, Martin LC, Almeida G. S, Matos MMM, Mainbourg MT, Franco RJS. Indians living in urban areas of Manaus, Amazon state, Brazil would be more protected to cardiovascular risk factors compared to their neighbors non-indians? In: ISN world congress of Nephrology 2013, Abstracts.

[26] Brasil (2014). Associação Brasileira de Empresas de Pesquisa (ABEP). Critério de Classificação Econômica Brasil.

[27] Lima CT, Freire ACC, Silva APB, Teixeira R, Farrell M, Prince M (2005). Concurrent and construct validity of the AUDIT in an urban Brazilian sample. Alcohol.

[28] Matsudo S, Araújo T, Matsudo V, Andrade D, Andrade E, Oliveira LC, Braggion G (2001). Questionário internacional de atividade física (IPAQ): estudo de validade e reprodutibilidade no Brasil. Rev Bras Ativ Fís Saude.

[29] Brasil. Associação Brasileira de Saúde Coletiva (ABRASCO). Inquérito Nacional de Saúde e Nutrição dos Povos Indígenas. Relatório Final: análise dos dados. n° 7. Rio de Janeiro, 2009.

[30] Ben-Noun L, Sohar E, Laor A (2001). Neck circumference as a simples screening measure for identifying over weight and obese patients. Obes Res.

[31] World Health Organization. Obesity: preventing and managing the global epidemic. Report of a World Health Organization consultation. Geneva: world health organization, 2000. 11234459

[32] Gallagher D, Heymsfield SB, Heo M, Jebb SA, Murgatroyd PR, Sakamoto Y (2000). Healthy percentage body fat ranges: an approach for developing guidelines based on body mass index. Am J Clin Nutr.

[33] OMRON. Manual de instruções: Balança de Controle Corporal (Balança de Bioimpedância) Modelo HBF-514C, 2014.

[34] O’Brien E, Mee F, Atkins N, Thomas M (1996). evaluation of three devices for self-measurement of blood pressure according to the revised British hypertension society protocol: the Omron HEM-705Cp, Philips HP5332, and Nissei DS-175. Blood Press Monit.

[35] Brasil. Diretrizes da Sociedade Brasileira de Diabetes. Tratamento e acompanhamento do Diabetes Mellitus. 2007:1–168.

[36] Xavier HT, Izar MC, Faria Neto JR, Assad MH, Rocha VZ, Sposito AC (2013). V Diretriz Brasileira de Dislipidemias e Prevenção da Aterosclerose. Arq Bras Cardiol.

[37] Brasil. Ministério da Saúde; Secretaria de Vigilância em Saúde. Vigitel Brasil 2015: Vigilância de fatores de risco e proteção para doenças crônicas por inquérito telefônico. Brasília: MS; 2016.

[38] Picon RV, Fuchs FD, Moreira LB, Riegel G, Fuchs SC (2012). Trends in prevalence of hypertension in Brazil: a systematic review with meta analysis. PLoS One.

[39] Fávaro T, Ribas DLB, Zorzatto JR, Segall-Corrêa AM, Panigassi G (2007). Food security in Teréna indigenous families, Mato Grosso do Sul, Brazil. Cad Saude Publica..

[40] Quermes PAA, Carvalho JA (2013). The impacts of assistance benefits on indigenous peoples: case study in Guarani settlements. Serv. Soc. Soc.

[41] Picon RV, Fuchs FD, Moreira LB, Fuchs SC (2013). Prevalence of hypertension among elderly persons in urban Brazil: a systematic review with meta-analysis. Am J Hypertens.

[42] Azevedo PS, Paiva SA, Zornoff LA (2014). Nutrition and cardiology: an interface not to be ignored. Arq Bras Cardiol.

[43] Pinho CPS, Diniz AS, Arruda IKG, Lira PIC, Sequeira LAS, Gonçalves FCLSP (2011). Overweight among adults in Pernambuco state, Brazil: prevalence and associated factors. Cad Saude Publica.

[44] Vedana EHB, Peres MA, Neves J, Rocha GC, Longo GZ (2008). Prevalence of obesity and potential causal factors among adults in southern Brazil. Arq Bras Endocrinol Metab.

[45] Rocha TES, Silva RP, Nascimento MM (2016). Changing dietary habits among Akwen Xerente. Rev Esc Enferm USP.

